# ^1^H, ^13^C, and ^15^N resonance assignments for the tandem PHD finger motifs of human CHD4

**DOI:** 10.1007/s12104-014-9582-y

**Published:** 2014-10-18

**Authors:** Louise J. Walport, Rosa Morra, Erika J. Mancini, Christina Redfield

**Affiliations:** 1 Department of Biochemistry, University of Oxford, South Parks Road, Oxford, OX1 3QU UK; 2 Division of Structural Biology, Henry Wellcome Building for Genomic Medicine, University of Oxford, Roosevelt Drive, Oxford, OX3 7BN UK

**Keywords:** Backbone resonance assignments, Plant homeodomain zinc finger, Heteronuclear NMR, Human chromodomain helicase DNA binding protein 4, Histones

## Abstract

The plant homeodomain (PHD) zinc finger is a structural motif of about 40–60 amino acid residues found in many eukaryotic proteins that are involved in chromatin-mediated gene regulation. The human chromodomain helicase DNA binding protein 4 (CHD4) is a multi-domain protein that harbours, at its N-terminal end, a pair of PHD finger motifs (dPHD) connected by a ~30 amino acid linker. This tandem PHD motif is thought to be involved in targeting CHD4 to chromatin via its interaction with histone tails. Here we report the ^1^H, ^13^C and ^15^N backbone and side-chain resonance assignment of the entire dPHD by heteronuclear multidimensional NMR spectroscopy. These assignments provide the starting point for the determination of the structure, dynamics and histone-binding properties of this tandem domain pair.

## Biological context

Plant homeodomain (PHD) fingers are short (~40–60 amino acid) protein domains which contain two zinc ions bound by a C4HC3 ligand motif with cross-brace ligation topology. They were discovered over 20 years ago (Schindler et al. 1993) and are found in more than 400 eukaryotic proteins, many of which are involved in the recognition of histone tails and in the chromatin remodelling mechanism (Mellor 2006). In recent years, a number of PHD motifs have been shown to act as specialized histone code “reader” modules that often recognize the methylation status of histone H3 tails (Pena et al. 2006; Shi et al. 2006; Wysocka et al. 2006; Zhang 2006). The biological importance of PHD fingers in human cells is highlighted by their occurrence in many chromatin-remodelling proteins linked to a variety of human diseases including cancer, mental retardation, and immunodeficiency (Baker et al. 2008).

**Fig. 1:**
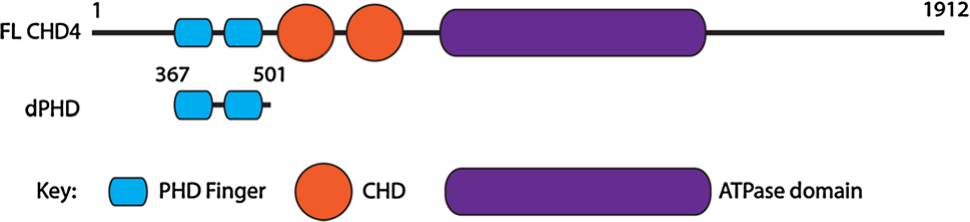
Domain architecture of CHD4. *Top* schematic of full length protein. *Bottom* schematic of dPHD construct assigned in this study

Chromodomain helicase DNA binding protein 4 (CHD4), also known as Mi2b, belongs to the SNF2 family of helicases (Eisen et al. 1995) and was first identified as a dermatomyositis-specific auto antigen (Seelig et al. 1995). CHD4 is the ATPase component of the nucleosome remodelling and deacetylases (NuRD) complex, which is involved in many repressive transcriptional regulatory processes (Ramirez and Hagman 2009). In addition to its SNF2-type ATPase motor, CHD4 harbours double PHD fingers (dPHD) and double chromodomains (dCHD) (Fig. 1) that are believed to be involved in its targeting to the chromatin (Morra et al. 2012), yet their mechanism of action is unclear. While the combination of PHD domains with other chromatin “reader” modules such as bromodomains is a feature of many chromatin-remodelling factors, the presence of tandem PHDs is characteristic of a much smaller subset of proteins including CHD4, CHD3, CHD5 and DPF3b. The structural mechanism of the acetylated histone binding by the double PHD fingers of DPF3b has been recently elucidated (Zeng et al. 2010) and represents the first solution structure of a tandem PHD finger domain pair. In the presence of an H3K14ac peptide, the PHD fingers of DPF3b act as a single functional unit with an extensive interface between the two PHD domains in which residues from both domains contribute to binding of a single peptide.

In human CHD4, the two PHD domains are separated by a 32 amino-acid linker, rich in acidic amino acids. This linker is significantly longer than that found in DPF3b. The length and sequence of the linker is well conserved in mammals, amphibians and bony fish. A linker of similar length and amino acid composition is also found in CHD3 and CHD5. Solution structures of the individual PHD1 and PHD2 of CHD4 have been reported and the mechanism of their binding to unmodified and methylated histone H3 has been described (Mansfield et al. 2011; Musselman et al. 2009). A more recent study has shown that together both PHDs of CHD4 can bind to nucleosomes in a multivalent manner, which is required for the repressive functioning of CHD4 (Musselman et al. 2012).

Here we report preliminary NMR studies of the tandem PHD fingers of human CHD4 and the complete ^1^H, ^13^C and ^15^N assignments. These serve as the starting point for the determination of the solution structure, dynamics and histone binding properties of dPHD which will help to elucidate the structural mechanism of histone H3 recognition by the entire tandem PHD unit of CHD4.

## Methods and experiments

### Protein expression and purification

The portion of the human CHD4 gene encoding the tandem PHD fingers (residues 367–501) was amplified by PCR using an appropriate set of primers. The amplified fragment was cloned in the IPTG-inducible pTriEx2 vector to generate a C-terminal 8xHis-tagged recombinant vector, which was used to transform Rosetta (DE3)pLysS *E. coli* cells. ^15^N and ^15^N/^13^C labelled recombinant proteins were expressed in the transformed cells grown in ^15^N-NH_4_Cl and ^13^C-glucose enriched M9 minimal medium supplemented with either 1 mM ZnSO_4_ or ZnCl_2_. The expressed proteins were first purified using a cobalt affinity column and eluted with a gradient of imidazole and then further purified by size exclusion chromatography in 20 mM Tris pH 7.5, 200 mM NaCl and 1–10 mM DTT.

### NMR spectroscopy

NMR samples contained ~1 mM protein in 95 % H_2_O/5 % D_2_O at pH 7.5 in 20 mM Tris with 200 mM NaCl and 10 mM DTT. All NMR spectra were acquired at 298 K using either home-built 750 or 950 MHz spectrometers which are controlled with GE/Omega software and are equipped with home-built triple-resonance pulsed-field-gradient probeheads or a Bruker Avance 500 MHz spectrometer with a Cryoplatform, equipped with a TCI CryoProbe. Sequential assignments were carried out initially using ^15^N-labelled dPHD and 3D ^15^N-edited TOCSY-HSQC (52 ms mixing time), NOESY-HSQC (150 ms mixing time) and HSQC-NOESY-HSQC (75 ms mixing time) experiments acquired at 950 MHz. Further backbone and side chain assignments were obtained using ^15^N- and ^13^C-labelled dPHD and 3D HNCA, HN(CO)CA, CBCANH, CBCA(CO)NH, HBHA(CBCACO)NH, H(CCO)NH, (H)CC(CO)NH, HNCO, HN(CA)CO and HCCH-TOCSY experiments acquired at 500 or 750 MHz.

## Extent of assignments and data deposition

**Fig. 2:**
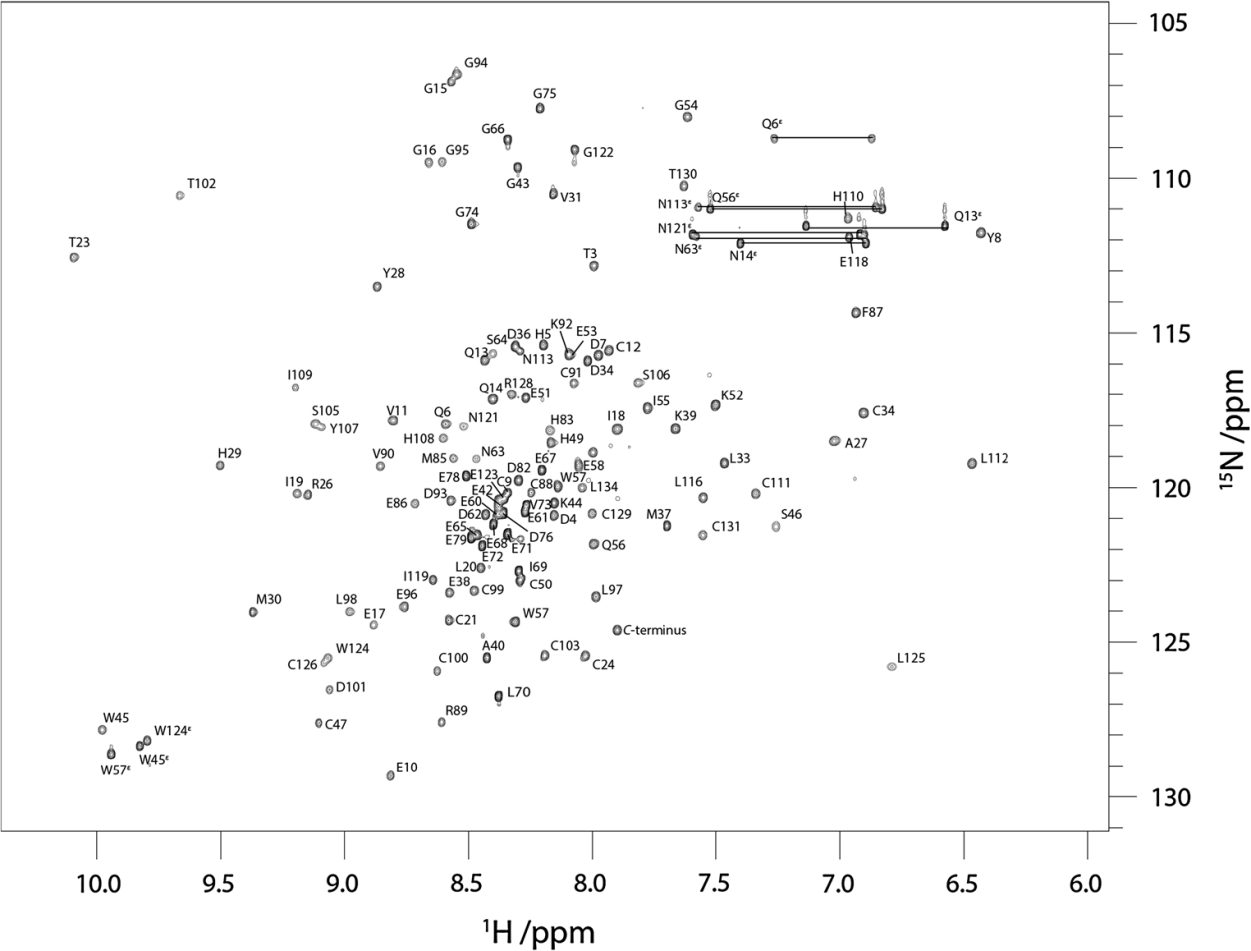
950 MHz ^1^H–^15^N HSQC spectrum of ^15^N-labelled dPHD at pH 7.5, 298 K. Peak assignments for backbone amides and the side chains of tryptophan, asparagine and glutamine are indicated. Residue 2 corresponds to residue 367 in the native sequence

Figure 2 shows the ^1^H–^15^N HSQC spectrum of dPHD collected at 950 MHz. Assigned backbone ^1^H^N^ and ^15^N are indicated. A total of 95 % of the backbone ^1^H^N^ and ^15^N of non-proline residues have been assigned. The side-chain ^1^H^N^–^15^N peaks for the three asparagine, four glutamine and three tryptophan residues are also labelled in Fig. 2. In addition, 95 % of the ^1^Hα, 93 % of the ^13^Cα, and 85 % of the ^13^C′ resonances were assigned. Most of the missing assignments correspond to the N-terminal residues, M1 and E2, three residues in the linker region, E80, E81 and H84, and the C-terminal residues G136 and K137, for which peaks in the ^1^H–^15^N HSQC spectrum could not be observed.

Extensive side-chain ^1^H and ^13^C assignments have also been made including 95 % of the ^1^Hβ and 94 % of the ^13^Cβ. For aliphatic residues, 87 % of the ^1^Hγ, 88 % of the ^13^Cγ, 68 % of the ^1^Hδ, 76 % of the ^13^Cδ, 44 % of the ^1^Hε and 36 % of the ^13^Cε have been assigned. For the Phe, Tyr and Trp residues, only aromatic ^1^H assignments have been made.

**Fig. 3:**
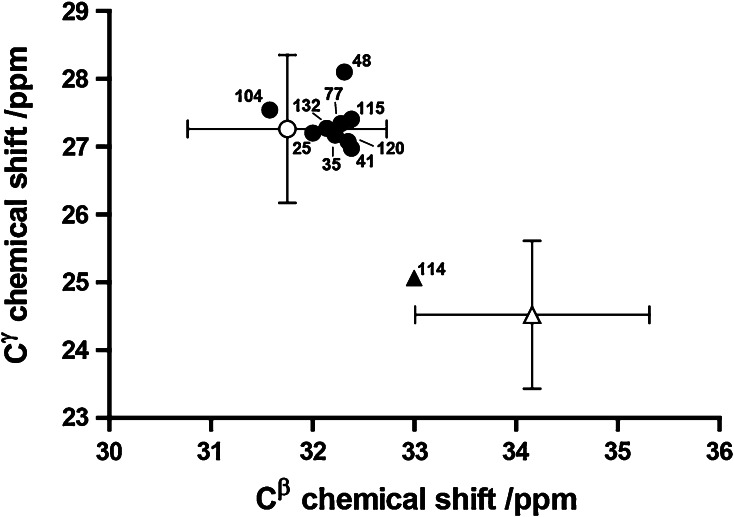
Analysis of proline ^13^Cβ and ^13^Cγ chemical shifts for dPHD. *Circles and triangles* correspond to residues with chemical shifts characteristic of a trans and cis conformation, respectively. The *open circle* and *open triangle* show the average chemical shifts reported (Schubert et al. 2002) for trans and cis proline, respectively; the *bars* indicate the standard deviation of these shifts

The ^13^Cβ and ^13^Cγ chemical shifts of ten of the twelve proline residues of dPHD have been analysed by the procedure of Schubert et al. (2002) to assign cis or trans peptide bond conformation (Fig. 3). We find that P114, located in the second PHD, is likely to adopt a cis conformation. This is confirmed by the ^1^Hα–^1^Hα NOE observed between N113 and P114. Interestingly, this residue is assigned a trans conformation in a previous structure determination by ^1^H NMR alone for the isolated 2nd PHD (Kwan et al. 2003).

The chemical shift assignments for dPHD have been deposited in the BioMagResBank (http://www.bmrb.wisc.edu) under the accession number 19968.

## References

[CR1] Baker LA, Allis CD, Wang GG (2008). PHD fingers in human diseases: disorders arising from misinterpreting epigenetic marks. Mutat Res.

[CR2] Eisen JA, Sweder KS, Hanawalt PC (1995). Evolution of the SNF2 family of proteins: subfamilies with distinct sequences and functions. Nucleic Acids Res.

[CR3] Kwan AHY, Gell DA, Verger A, Crossley M, Matthews JM, Mackay JP (2003). Engineering a protein scaffold from a PHD finger. Structure.

[CR4] Mansfield RE, Musselman CA, Kwan AH, Oliver SS, Garske AL, Davrazou F, Denu JM, Kutateladze TG, Mackay JP (2011). Plant homeodomain (PHD) fingers of CHD4 are histone H3-binding modules with preference for unmodified H3K4 and methylated H3K9. J Biol Chem.

[CR5] Mellor J (2006). It takes a PHD to read the histone code. Cell.

[CR17] Morra R, Lee BM, Shaw H, Tuma R, Mancini EJ (2012) Concerted action of the PHD, chromo and motor domains regulates the human chromatin remodelling ATPase CHD4. FEBS Lett 586:2513–2521 10.1016/j.febslet.2012.06.017 PMC3476528 22749909

[CR6] Musselman CA, Mansfield RE, Garske AL, Davrazou F, Kwan AH, Oliver SS, O’Leary H, Denu JM, Mackay JP, Kutateladze TG (2009). Binding of the CHD4 PHD2 finger to histone H3 is modulated by covalent modifications. Biochem J.

[CR7] Musselman CA, Ramirez J, Sims JK, Mansfield RE, Oliver SS, Denu JM, Mackay JP, Wade PA, Hagman J, Kutateladze TG (2012). Bivalent recognition of nucleosomes by the tandem PHD fingers of the CHD4 ATPase is required for CHD4-mediated repression. Proc Natl Acad Sci USA.

[CR8] Pena PV, Davrazou F, Shi XB, Walter KL, Verkhusha VV, Gozani O, Zhao R, Kutateladze TG (2006). Molecular mechanism of histone H3K4me3 recognition by plant homeodomain of ING2. Nature.

[CR9] Ramirez J, Hagman J (2009). The Mi-2/NuRD complex: a critical epigenetic regulator of hematopoietic development, differentiation and cancer. Epigenetics.

[CR10] Schindler U, Beckmann H, Cashmore AR (1993). HAT3.1, a novel Arabidopsis homeodomain protein containing a conserved cysteine-rich region. Plant J.

[CR11] Schubert M, Labudde D, Oschkinat H, Schmeider P (2002). A software tool for the prediction of Xaa-Pro peptide bond conformations in proteins based on ^13^C chemical shift statistics. J Biomol NMR.

[CR12] Seelig HP, Moosbrugger I, Ehrfeld H, Fink T, Renz M, Genth E (1995). The major dermatomyositis-specific Mi-2 autoantigen is a presumed helicase involved in transcriptional activation. Arthritis Rheum.

[CR13] Shi X, Hong T, Walter KL, Ewalt M, Michishita E, Hung T, Carney D, Pena P, Lan F, Kaadige MR, Lacoste N, Cayrou C, Davrazou F, Saha A, Cairns BR, Ayer DE, Kutateladze TG, Shi Y, Cote J, Chua KF, Gozani O (2006). ING2 PHD domain links histone H3 lysine 4 methylation to active gene repression. Nature.

[CR14] Wysocka J, Swigut T, Xiao H, Milne TA, Kwon SY, Landry J, Kauer M, Tackett AJ, Chait BT, Badenhorst P, Wu C, Allis CD (2006). A PHD finger of NURF couples histone H3 lysine 4 trimethylation with chromatin remodelling. Nature.

[CR15] Zeng L, Zhang Q, Li S, Plotnikov AN, Walsh MJ, Zhou MM (2010). Mechanism and regulation of acetylated histone binding by the tandem PHD finger of DPF3b. Nature.

[CR16] Zhang Y (2006). It takes a PHD to interpret histone methylation. Nat Struct Mol Biol.

